# Treatment of Terminal Syrinx With a Syringo-Subarachnoid Shunt and the Novel Use of a Titanium Knot Fastener: A Case Report

**DOI:** 10.7759/cureus.73440

**Published:** 2024-11-11

**Authors:** Taylor Furst, Jayden Allegakoen, Muhammad I Jalal, Rohin Singh, Jonathan J Stone

**Affiliations:** 1 Neurological Surgery, University of Rochester Medical Center, Rochester, USA

**Keywords:** cor-knot micro, csf diversion procedures, syringo-subarachnoid shunt, terminal syrinx, titanium fastener

## Abstract

A syrinx involves cystic dilation of the central canal of the spinal cord due to the accumulation of cerebrospinal fluid and often results in a neurological deficit. While treatment options vary, surgical management is often utilized and requires an open durotomy.

A 70-year-old female presented with one year of progressive low back pain with associated leg numbness, urinary incontinence, bilateral foot drop, and imbalance resulting in multiple falls. MRI revealed a terminal syrinx at the level of the conus medullaris. She underwent an uncomplicated T12-L1 laminectomy for syrinx fenestration and syringo-subarachnoid shunt placement, resulting in improved bladder/bowel function, strength, sensation, and ambulation. The necessary midline durotomy was closed with a running 6-0 prolene suture fastened at the start and end of the suture line with the novel use of the COR-KNOT MICRO^TM^ device (LSI Solutions, Victor, New York, US).

We present the successful surgical management of a progressively symptomatic terminal syrinx using a syringo-subarachnoid shunt and the novel use of the COR-KNOT MICRO^TM^ titanium fastener device for dural closure. Earlier shunting should be considered in the disease course of terminal syrinx, and the successful novel use of a titanium fastener on the dura warrants further investigation.

## Introduction

A syrinx is the formation of a cerebrospinal fluid (CSF) filled cavity within the spinal cord parenchyma or central canal, often associated with occult spinal abnormalities, and is a rare spinal pathology that can be difficult to treat [1]. While a syrinx is often associated with Chiari malformations, it can also result from other etiologies such as trauma, infection, degenerative changes, neoplasm, or idiopathic [2]. Terminal syrinx (TS), or a syrinx in the distal third of the spinal cord, is one such example. The clinical manifestations of TS are often sensory disturbances but the presentation can also include signs of myelopathy such as imbalance, weakness, and hyperreflexia due to spinal cord and conus medullaris dysfunction. These symptoms can be devastating, leading to poor quality of life from falls with injury and inability to work leading to financial constraints. Magnetic resonance imaging (MRI) is most often employed to confirm diagnosis, and treatment varies. TS resulting in mild symptoms is typically managed conservatively with clinical surveillance, though severe or progressively symptomatic cases often require surgical management with open durotomy for fenestration or shunt placement [2,3]. Specifically, CSF shunting of the syrinx, most commonly to the subarachnoid space, is often reserved for progressive and recurrent disease [4,5].

Here, we present a case of a progressively symptomatic idiopathic TS successfully managed with upfront syringo-subarachnoid (SS) shunting and the novel use of the COR-KNOT MICRO^TM^ titanium fastener device (LSI Solutions, Victor, New York, US) for dural closure. The purpose of this case report is to report the successful early use of SS shunting and the successful novel use of a titanium fastener to secure suture on dura.

## Case presentation

Initial presentation

**Figure 1:**
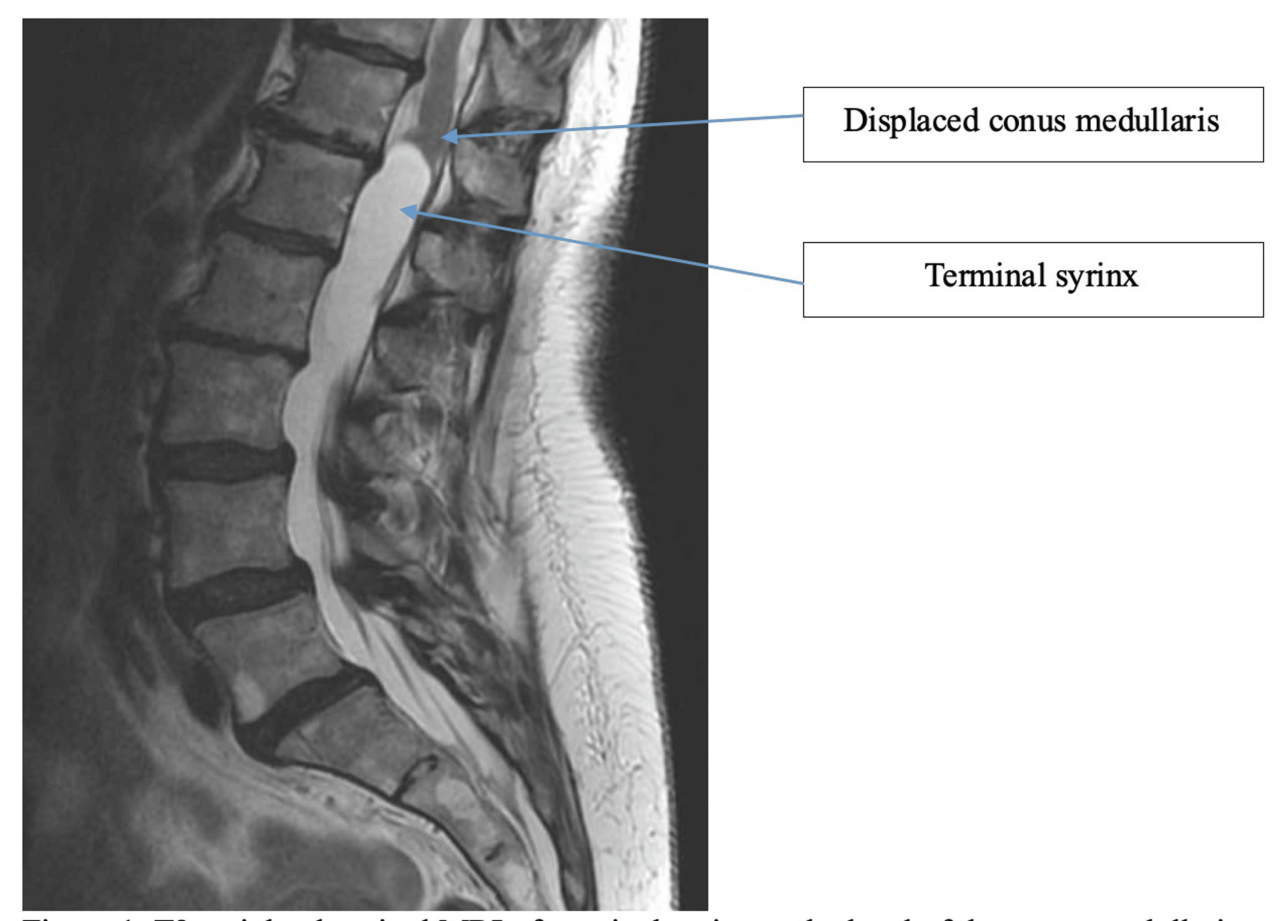
T2-weighted sagittal lumbar spine MRI of the terminal syrinx at the level of the conus medullaris The arrow points to the displaced conus medullaris and the terminal syrinx.

A 70-year-old female presented with one year of progressive bilateral foot drop, lower extremity (LE) numbness, back pain, myelopathic imbalance with recurrent falls, and six months of urinary incontinence. Past medical history was non-contributory, and she had no prior spine surgery. Physical exam revealed a Babinski reflex in the right LE, bilateral foot drop with 1-2/5 strength, sensation loss in the bilateral LEs, and an unsteady steppage gait. MRI of the lumbar spine was notable for a TS at the level of the conus medullaris without extrinsic compression or significant stenosis (Figure 1).

The operative technique

Via a standard posterior approach, a T12-L1 laminectomy with midline durotomy for syrinx fenestration and SS shunt placement was performed in an uncomplicated fashion. A minimally invasive approach was considered; however, it was felt to limit necessary neural tissue visualization, identification of a safe syrinx entry point in neural tissue, and durotomy closure. Thus, a standard open posterior approach was felt to be more likely to allow safe and successful SS placement, avoid a postoperative neurological deficit, and achieve symptomatic improvement. Intraoperative neuro-monitoring was utilized in the form of motor-evoked potentials, sensory-evoked potentials, and electromyography. Following laminectomy, an ultrasound was placed on the dura to localize the syrinx and guide the site of durotomy.

**Figure 2:**
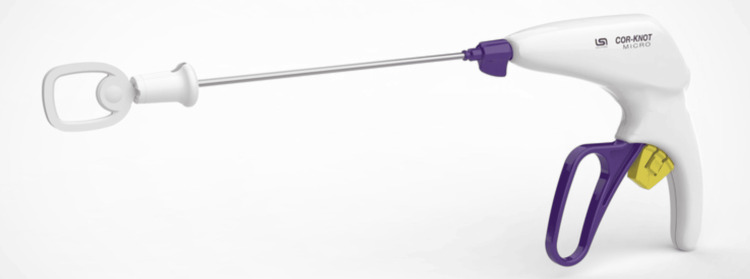
LSI COR-KNOT MICRO titanium fastener device The picture and a description of the LSI COR-KNOT MICRO titanium fastener device are publicly available at: https://www.lsisolutions.com/products/10/cor-knot-micro/. COR-KNOT MICRO bolsters a rotational 14 cm shaft that is 3 mm in diameter. The suture is manually threaded through the wire snare at the end of the device to load the suture into the embedded titanium fastener. The purple handle is then squeezed, which secures the suture by crimping the titanium fastener around the suture while also trimming its ends. The titanium fastener is MRI-compatible up to 3 tesla. LSI Solutions, Victor, New York, US

After a midline dorsal durotomy was created, neuromonitoring confirmed no response at the planned myelotomy site for syrinx fenestration and shunt placement. Upon entering the syrinx, rapid spontaneous egress of pressurized CSF was noted. The proximal end of the shunt catheter was passed into the syrinx cavity while the distal end was secured to the dura in the subarachnoid space. The durotomy was then closed using a 6-0 Prolene running suture. The COR-KNOT MICRO^TM^ titanium fastener device was secured on both ends of the suture line instead of hand-tied (HT) knots (Figure 2). First, the device is loaded by threading the suture through its shaft, thereby also threading it through the preloaded titanium fastener. The operator then places the end of the device at the site of the intended knot. Counter-tension is applied on the suture ends by the operator to the tension of their choice. Once the site of placement and tension is deemed acceptable by the surgeon, the trigger is pulled, securing the suture by crimping the titanium fastener around the suture and cutting the residual ends. Following this process, Valsalva confirmed no CSF leak from the durotomy site, thus no on-lay product was utilized. The wound was closed in a standard layered fashion, including fascia, subcuticular tissues, and skin. Video 1 provides a visual representation of shunt placement and the use of the COR-KNOT MICRO^TM^ device for dural closure.

**Video 1:** Video case presentation Demonstrating posterior midline open spine exposure followed by a bilateral laminectomy, midline dorsal durotomy creation, syringo-subarachnoid shunt placement, and finally dural closure utilizing the COR-KNOT MICRO titanium fastener device LSI Solutions, Victor, New York, US

Postoperative course

**Figure 3:**
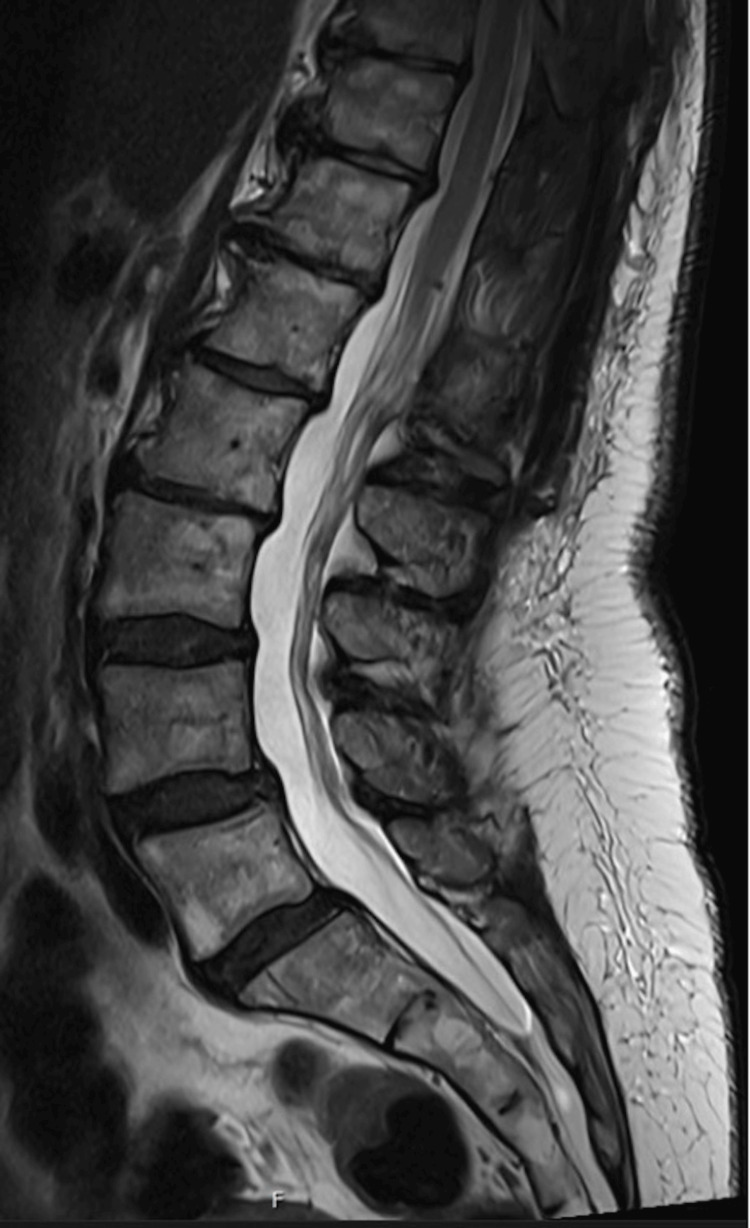
Postoperative T2-weighted sagittal MRI Postoperative T2-weighted sagittal MRI with resolved terminal syrinx and lack of artifact from the titanium fastener

Immediately postop, the patient reported improved LE numbness, and she was discharged home on postop day 3 after clearing inpatient physical therapy (PT). At two weeks postop, she reported improved urinary incontinence and LE strength (i.e., resolved left foot drop) in addition to ongoing improvement in LE sensation. With ongoing PT, at three months postop, she now also reported improved balance and ambulation with no further falls and felt her preoperative symptoms were overall 80% resolved. MRI at one year postop showed resolution of the TS and no significant artifact from the titanium fasteners (Figure 3).

## Discussion

TS remains an uncommon and challenging pathology to manage. Management varies from surveillance to CSF diversion depending upon the degree of neurological impairment [1,2]. Simple decompression is insufficient, as TS, in this case, was not associated with Chiari malformation and was not due to extrinsic compression from spinal column elements such as spondylotic changes. Here, we describe our experience treating a TS causing significant progressive myelopathy. By using SS shunting, we achieved successful resolution of the TS on postoperative imaging and significant neurologic improvement consistent with prior reports of CSF diversion for the syrinx [4-6]. While successful conservative management of TS can be seen, shunting is often needed especially with progressive or refractory symptoms [5,7,8]. We report the successful deployment of this SS shunting in the setting of progressive and severely symptomatic TS. This success builds upon evidence in current literature that SS shunting is safe and effective [5,7,8].

In addition, we also report the successful and novel use of the LSI COR-KNOT MICRO^TM^ device for dural closure. To the best of our knowledge, this is the first use of this technology for dural closure. This device is FDA-approved for the closure of soft tissues and provides a means for automated knot tying. It utilizes a titanium fastener crimped to suture ends in place of HT knots and has been shown to secure the suture in a stronger, more consistent, and faster way when directly compared to HT knots [9-13]. The device achieves these feats by crimping the titanium fastener to the suture and then cutting the suture ends with just one squeeze of the device's trigger. Though the use of this technology for dural closure is novel, its successful ability to facilitate closure is predicted by its efficacy in other fields, namely, laparoscopic general surgery as well as coronary anastomosis and valve replacements during cardiac surgery [9-13].

The outcome of this case suggests that the use of SS shunts for the syrinx should be considered earlier in the disease course rather than reserved for recurrent disease. Furthermore, the successful deployment of COR-KNOT MICRO^TM^ for dural closure foreshadows its potential to be adapted for dedicated use on the dura, both in open and minimally invasive approaches. The lack of artifact from the titanium fastener on MRI is critical for accurate postoperative evaluation bolstering the possibility of expanded use of this device on dura mater. It is important to note that this case is only one example of SS shunting for TS and dural closure with COR-KNOT MICRO^TM^. Results should be interpreted with caution, though they warrant further investigation.

## Conclusions

This case presents the successful surgical management of a TS with early SS shunting. Additionally, this case represents the first time that the COR-KNOT MICRO^TM^ titanium fastener technology has been used for dural closure. SS shunting resulted in significant neurological recovery with no intraoperative nor postoperative complications in addition to the resolution of the TS on postoperative imaging. The use of this titanium fastener device for dural closure resulted in no CSF leak, an artifact on postoperative imaging, or complications at the one-year follow-up. Earlier employment of SS shunting for TS and the use of the COR-KNOT MICRO^TM^ titanium fastener device on dura should be considered for further investigation, as this patient experienced significant neurological improvement and no CSF leak was present at the one-year follow-up.
